# Patient-reported and clinical outcomes after first-time atrial fibrillation ablation in older patients: a real-world retrospective single-center study

**DOI:** 10.3389/fcvm.2026.1841603

**Published:** 2026-07-08

**Authors:** Pernille Borch, Ole-Gunnar Anfinsen, Finn Hegbom, Knut Sevre, Torbjørn Holm, Trine Synnøve Fink, Lars Andreas Dejgaard, Erik Kongsgård, Mathis Korseberg Stokke, Erik Lyseggen

**Affiliations:** 1 Institute of Clinical Medicine, University of Oslo, Oslo, Norway; 2 Department of Cardiology, Oslo University Hospital Rikshospitalet, Oslo, Norway; 3 Institute for Experimental Medical Research, Oslo University Hospital and University of Oslo, Oslo, Norway

**Keywords:** atrial fibrillation, catheter ablation, older patients, patient-reported outcome measures (PROMs), symptom management

## Abstract

**Background and aims:**

Older patients remain underrepresented in studies of catheter ablation (CA) for atrial fibrillation (AF), and patient-reported outcome measures (PROMs) after ablation in this population are limited. We compared real-world clinical outcomes and PROMs after first-time CA for AF in patients ≥70 years and 60–65 years.

**Methods:**

All patients aged ≥70 or 60–65 years undergoing first-time CA for AF at Oslo University Hospital Rikshospitalet between 2017 and 2021 were retrospectively invited in 2022 to complete standardized questionnaires. The modified European Heart Rhythm Association (mEHRA) classification was used to grade symptoms, with change expressed as *Δ*mEHRA (follow-up minus baseline). Clinical and procedural data were retrieved from medical records.

**Results:**

Of 462 eligible patients, 344 were included: 132 ≥ 70 years (median age 73.0, 64% male), and 212 aged 60–65 (median age 63.0, 73% male). Both groups reported significant symptom relief one year after ablation (≥70: ΔmEHRA −1.42 ± 1.29, *p* < 0.001; 60–65: ΔmEHRA −1.78 ± 1.17, *p* < 0.001), with greater absolute reduction in the younger group in unadjusted analyses (*p* = 0.021). At follow-up, median mEHRA scores were low and identical between groups [1 (1–2) vs. 1 (1–2)], with a greater proportion of older patients reporting any improvement (*p* = 0.034). Complication rates were not statistically significantly different (5% vs 7%, *p* = 0.62), whereas reported AF recurrence was higher among older patients (59% vs 47%, *p* = 0.042).

**Conclusion:**

In this real-world cohort, patients ≥70 years undergoing first-time CA for AF experienced meaningful symptom improvement and no statistically significant difference in complication rates compared with patients aged 60–65 years, despite higher reported recurrence rates. These findings highlight the value of PROMs alongside conventional rhythm outcomes when assessing treatment benefit in older patients.

## GRAPHICAL ABSTRACT

**Figure:**
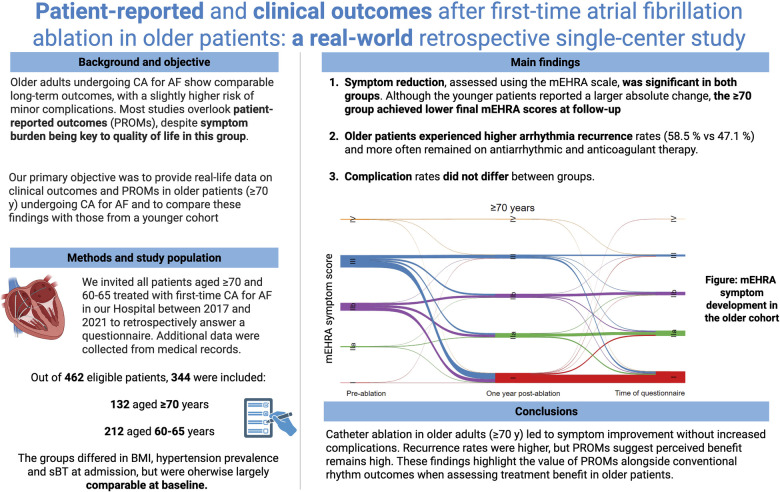
Created in BioRender. BORCH, P. (2026) https://BioRender.com/azl9ggr.

## 1 Introduction

The global prevalence of atrial fibrillation (AF) continues to increase, largely driven by population aging and the steep rise in AF incidence with advancing age (1). Older patients constitute a substantial portion of the AF population, yet they remain underrepresented in studies of catheter ablation (CA) (1). Previous systematic reviews and meta-analyses suggest that procedural complications may be modestly higher in older patients, whereas freedom from arrhythmia outcomes appear largely comparable across age groups (2–5). However, interpretation is limited by heterogeneity in age cutoffs, follow-up strategies and endpoint definitions (5). Additional data from routine clinical practice may therefore be useful to inform treatment decisions in this growing patient population.

Among older patients with AF, symptom severity has been shown to be the strongest determinant of health-related quality of life (6). Because the experience of AF and its treatment is inherently subjective, patient-reported outcome measures (PROMs) should be incorporated as core endpoints in clinical trials, both to guide patient-centered care and to provide a fuller assessment of therapeutic efficacy (7). Yet most studies on effects of CA in older patients have focused on recurrence rates and complications (2–5), whereas PROM-based data remain limited.

Our primary objective was to compare real-world PROMs and clinical follow-up after first-time CA for AF in patients ≥70 years with a younger reference cohort representative of older segments of patients included in previous AF ablation trials.

## 2 Methods

### 2.1 Approvals

This study was approved by the Norwegian Regional Committee for Medical and Health Research Ethics (REK) (22/487169) and the Data Protection Officer at Oslo University Hospital Rikshospitalet (22/19128). Written informed consent was obtained from all participants alive at the time of data collection prior to study inclusion. Deceased patients were automatically included.

### 2.2 Patient population

In this retrospective, single-center study we invited all patients aged ≥70 years or 60–65 years who had been treated with first-time pulmonary vein isolation in our tertiary referral hospital between January 2017 and December 2021. No additional exclusion criteria were applied within the selected age groups.

*The older cohort* comprised patients aged ≥70 years, consistent with many AF-ablation studies and reflecting an age range in which left-atrial remodeling, comorbidity burden, and procedural risk become increasingly relevant. *The younger cohort* comprised patients aged 60–65 years, i.e., just below the CHA₂DS₂-VASc age threshold (≥65 years), while still representing the upper age range commonly included in major AF ablation trials (8).

### 2.3 Data collection

In 2022, patients were asked to complete standardized questionnaires and provide consent for the use of their medical records for data collection. Data were collected from electronic patient records and questionnaires distributed to all eligible patients along with the study invitation (Supplementary Material S1). Patients were also given the option to respond to the questionnaire via a structured telephone interview. Questionnaire responses were cross-checked against medical records, with discrepancies resolved in favor of the latter. Parameters extracted from the medical records included clinical and procedural characteristics.

In case of missing questionnaire responses, patients who had consented to being contacted were called to clarify their answers. Deceased patients contributed data from medical records only, as questionnaire-based PROM data were unavailable. Remaining missing data were not imputed; analyses were performed using available data for each variable.

Data collection took place between January 2023 and June 2024, corresponding to a follow-up period ranging from one to seven and a half years after the patient's initial ablation procedure.

### 2.4 Clinical characteristics

Clinical characteristics included age, sex, height, weight, body mass index (BMI), AF type and duration, hypertension, diabetes mellitus, systolic and diastolic blood pressure at admission. Additionally, histories of vascular disease, coronary artery disease, myocardial infarction, percutaneous coronary intervention or coronary artery bypass grafting, peripheral artery disease, prior stroke or transient ischemic attack and congenital heart disease were recorded. Further comorbidity data included New York Heart Association class, CHA₂DS₂-VASc score, cardiomyopathy, dyslipidemia, chronic obstructive pulmonary disease, pulmonary hypertension and kidney disease.

Information on the presence of implantable devices (pacemaker, cardiac resynchronization therapy, implantable cardioverter-defibrillator), sleep apnea, atrial flutter, tachycardia-induced heart failure, and pro-brain natriuretic peptide levels were also collected, along with presence of other comorbidities. The use of antiarrhythmic drugs (AADs) and anticoagulation was documented.

Echocardiographic and imaging parameters at admission were collected, including ejection fraction (EF%), left ventricular end-diastolic diameter, left atrial diameter, left atrial area, left atrial indexed volume, right atrial area, presence and severity of mitral valve insufficiency or other valvular diseases, and the performance of additional procedures such as coronary angiography and cardiac magnetic resonance imaging.

### 2.5 Questionnaires for patient-reported outcomes

Our questionnaire comprised questions on complications, recurrence, necessity for follow-up and/or additional CA procedures, symptom severity and burden, as well as medications prior to, one year after CA, and at the time of the questionnaire (Supplementary Material S1). Patients who underwent several CA procedures during the study period were asked to answer one questionnaire for each procedure. Symptom severity was categorized according to the validated modified European Heart Rhythm Association (mEHRA) symptom classification in AF. The remaining questionnaire items were developed in collaboration with senior researchers in the department, as no existing validated instrument fully addressed the specific follow-up questions relevant to this study. Several items had previously been used in routine structured follow-up one year after ablation at our center for quality control purposes and were therefore considered clinically relevant and feasible for this retrospective evaluation. Arrhythmia burden was assessed using a five-point scale based on the duration and termination of AF episodes: 0 = no AF episodes; 1 = self-terminating episodes lasting <24 h; 2 = self-terminating episodes lasting >24 h; 3 = termination requiring pharmacological cardioversion; 4 = termination requiring electrical cardioversion; and 5 = chronic AF, indicating a shared decision between the patient and physician to forgo further attempts at rhythm control.

### 2.6 Procedures, acute outcomes and complications

Procedural parameters included the year of CA, total procedure time, ablation type [radiofrequency (RF) or cryoablation], concurrent cavotricuspid isthmus block, use of anesthesia, radiation time and dose, number of RF applications, RF/cryo application time, acute procedural success, complications, and their respective management.

Ablation modality was selected by the operator according to clinical judgement and prevailing practice during the study period. In older patients, radiofrequency ablation was often preferred when a more detailed electroanatomical evaluation of the left atrium was considered clinically useful, including assessment of atrial substrate and low-voltage areas, and to facilitate individualized planning in case of future repeat ablation. Radiofrequency procedures were performed according to a strict CLOSE-guided protocol.

### 2.7 Endpoints

The primary endpoint of this study was patient-reported symptom severity before and after CA, assessed using questions based on the mEHRA symptom score, and arrhythmia burden was assessed using a patient-reported score from 1 to 5 based on duration of AF episodes and potential need for medical or electrical cardioversion to terminate the episodes.

Secondary endpoints included recurrence and complication rates, need for another CA procedure for AF, and antiarrhythmic and anticoagulant use. Recurrence was defined as either patient-reported experience of AF symptoms after a three-month blanking period post ablation, or recurrence reported as documented in medical records. Complications and medications were assessed based on the responses to the questionnaires and medical records.

### 2.8 Statistical analysis

Patient demographics, medical history characteristics, and medications were summarized using mean and standard deviation (SD) for normally distributed continuous variables, and median with interquartile range (IQR) for non-normally distributed data. Normality was assessed using the Shapiro–Wilk test. Categorical variables were reported as frequencies and percentages. Group comparisons were performed using the independent two-sample *t*-test for normally distributed continuous variables and the Mann–Whitney *U*-test for non-normally distributed continuous and ordinal variables. Categorical variables were compared using Pearson's *χ*^2^ test for variables with ≥5 observations in each category, and Fisher's exact test for variables with <5 observations in one or more categories. Changes in non-normally distributed ordinal variables within groups were tested using Wilcoxon signed-rank test. Changes in binary variables within groups were assessed using McNemar's *χ*^2^ test. A *p*-value of <0.05 was considered statistically significant.

Between-group analyses were unadjusted and considered exploratory, and *p*-values should be interpreted without formal correction for multiple comparisons.

An exploratory Spearman correlation analysis was performed to assess whether symptom change was associated with ablation year. Time from index ablation to questionnaire completion was approximated using ablation year, coded from 2017 to 2021, as all questionnaires were completed in 2022.

## 3 Results

### 3.1 Patient population

**Figure 1:**
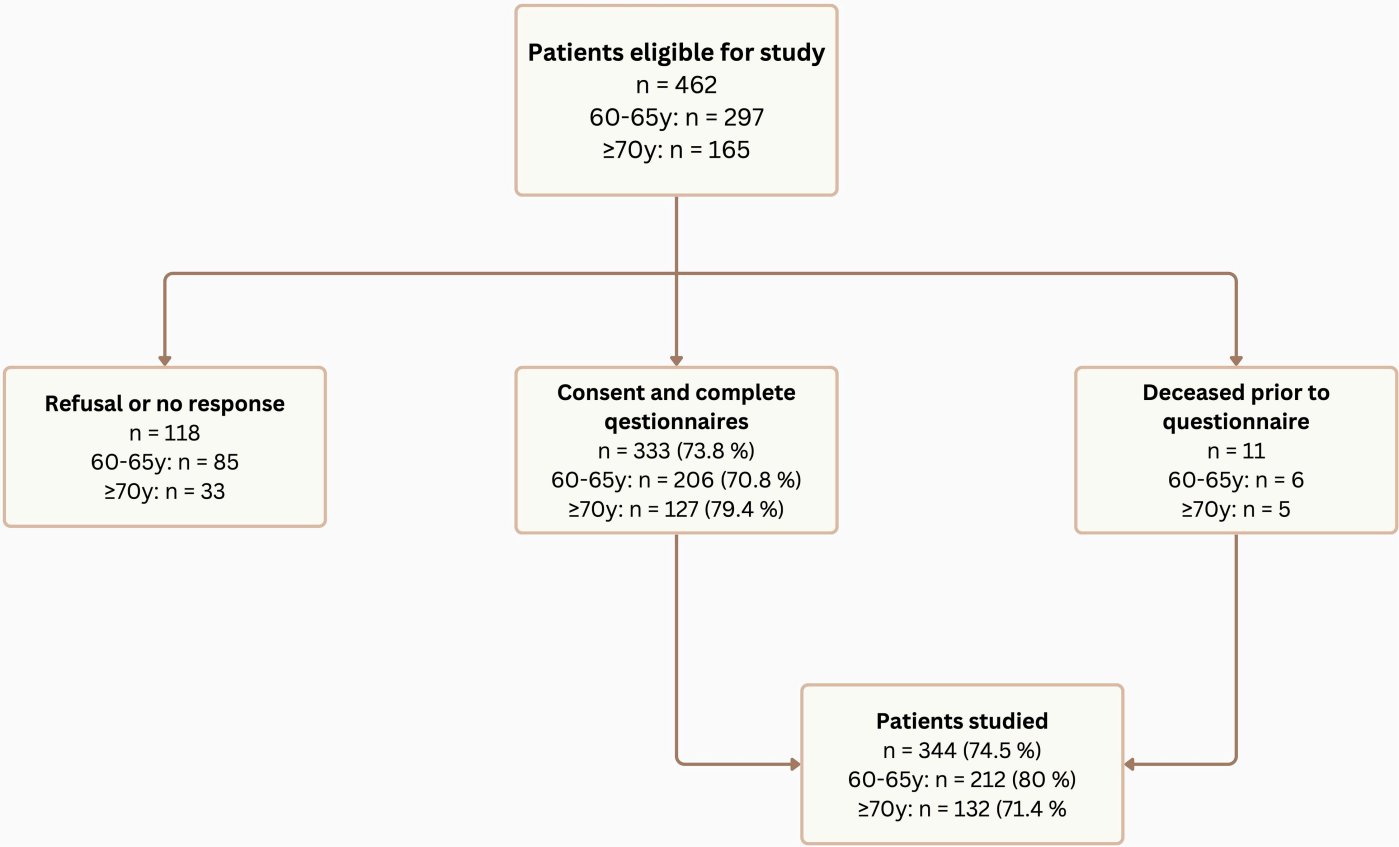
Flowchart for inclusion.

Out of 462 eligible patients, 344 were included in the study; 132 in the older cohort (median age 73.0, 64% male), and 212 in the younger cohort (median age 63.0, 73% male) (Figure 1). Eleven patients were deceased at study start, and were included based on available data from medical records only.

Clinical characteristics for the two groups were compared (Table 1, Supplementary Table S2). Patients in the younger cohort were taller, heavier and had higher BMI than patients in the older cohort (Table 1, Supplementary Table S2). CHA_2_DS_2_VA-score was significantly lower in the younger cohort, however not when excluding the points for age from the score. There were more patients with a history of hypertension in the younger cohort, but systolic blood pressure on admission was significantly lower in this group. Finally, there was a lower percentage of valvular disease excluding mitral regurgitation in the younger cohort compared to the older. Other clinical parameters collected were similar between the two groups.

**Table 1:** Baseline characteristics.

	≥70 years (*N* = 132)		60–65 years (*N* = 212)		
Characteristic	n	Value	n	Value	*p*-value
Age at procedure, years	132	73.0 (71.0–75.0)	212	63.0 (61.0–64.0)	<0.001*
Gender, Male (%)	132	85 (64)	212	154 (73)	0.11
BMI (SD)	131	26.0 (3.4)	211	27.6 (3.8)	<0.001*
Type of AF	131		209		0.15
Paroxysmal (%)		95 (72)		168 (79)	0.12
Paroxysmal persistent (%)		6 (5)		11 (5)	0.79
Persistent (%)		28 (21)		24 (11)	0.13
Long-standing persistent (%)		2 (2)		6 (3)	0.72
AF duration, years (IQR)	118	4.0 (2.0–9.0)	191	3.0 (1.2–7.0)	0.035
CHA2DS2VA (IQR)	132	2.0 (1.0–3.0)	212	1.0 (0.0–1.0)	<0.001*
Systolic BP at admission, mm Hg (IQR)	125	140.0 (131.0–159.0)	197	135.0 (121.0–150.0)	0.002*
Diastolic BP at admission, mm Hg (IQR)	125	79.0 (71.0–85.0)	197	78.0 (72.0–84.0)	0.96
EF % (IQR)	75	55.0 (50.0–60.0)	115	55.0 (51.0–60.0)	0.74
LVEDD, cm (SD)	132	5.1 (0.5)	204	5.2 (0.7)	0.28
LAD, cm (IQR)	56	4.2 (3.8–4.7)	94	4.4 (3.9–4.8)	0.63
LAA, cm^2^ (IQR)	128	24.2 (21.0–28.0)	196	24.4 (21.0–28.0)	0.70
LAVI, mL/m^2^ (IQR)	90	40.5 (32.0–48.0)	131	37.0 (31.0–47.0)	0.19
RAA, cm^2^ (IQR)	71	20.0 (16.0–23.0)	100	20.0 (17.0–24.0)	0.39
MR (%)	130	66 (51)	206	83 (40)	0.071
Other valve disease (%)	132	13 (10)	207	7 (3)	0.014*
Use of antiarrhythmics (%)	132	119 (90)	212	186 (88)	0.49
Use of anticoagulants (%)	132	131 (99)	212	207 (98)	0.270

AF, atrial fibrillation; BMI, body mass index; BP, blood pressure; CHA2DS2VA, Congestive heart failure, Hypertension, Age ≥75 years (doubled), Diabetes mellitus, prior Stroke or TIA or thromboembolism (doubled), Vascular disease, Age 65 to 74 years; EF, ejection fraction; LAA, left atrial area; LAD, left atrial diameter; LAVI, left atrial volume index; LVEDD, left ventricular end-diastolic diameter; MR, mitral valve regurgitation; RAA, right atrial area. Detailed baseline characteristics are presented in Supplementary Table S2.

* *p* < 0.05.

The adjacent n columns indicate the number of patients with available data for each variable. Continuous variables are presented as median (IQR) or mean (SD), and categorical variables as n (%) or n, as indicated.

### 3.2 Self-reported symptom severity

**Figure 2:**
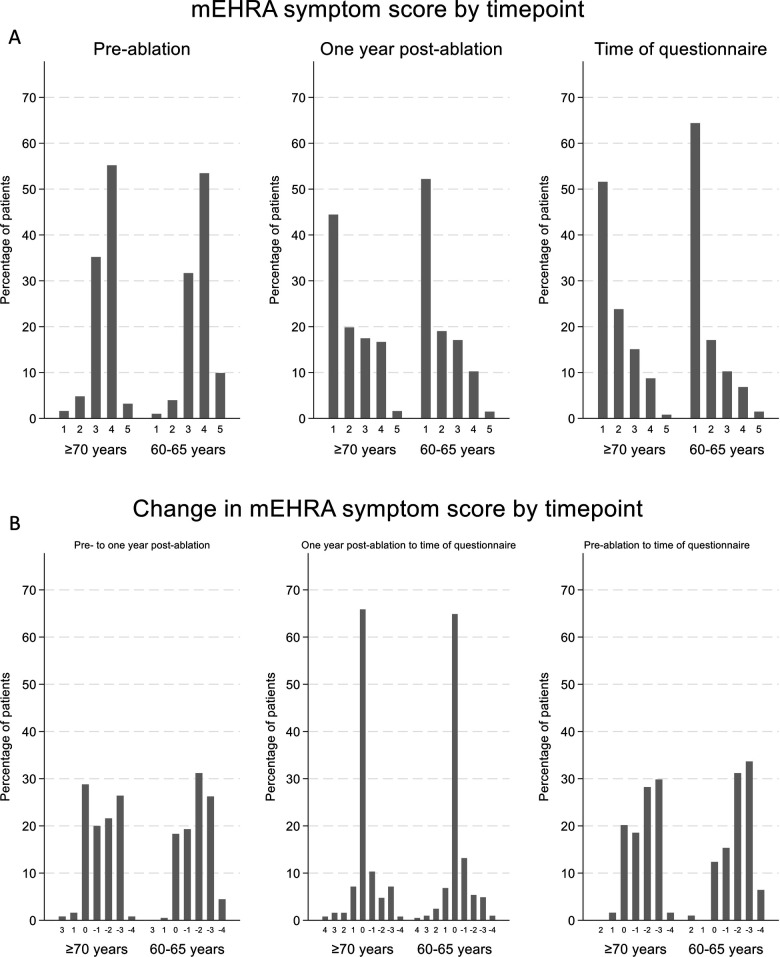
**(A)** Distribution of patient-reported mEHRA scores by age at each timepoint. **(B)** Change in mEHRA scores by age at each timepoint. Example: a value of −2 may represent a change from a score of 4 to a score of 2.

Mean time to follow-up was 44.5 ± 16.3 months. Self-reported symptom severity at different time points relative to CA was assessed using the mEHRA symptom scale. Scores did not differ significantly between age cohorts at baseline [4 (3–4) vs. 4 (3–4), *p* = 0.13] or at one-year post-ablation [2 (1–3) vs. 1 (1–3), *p* = 0.11] (Figure 2A). At the time of the questionnaire, median mEHRA scores were low and identical in the two groups [1 (1–2) vs. 1 (1–2)], while a greater proportion of patients in the older cohort reported improvement relative to baseline (Rank sum with Mann–Whitney *U*, *p* = 0.034).

**Figure 3:**
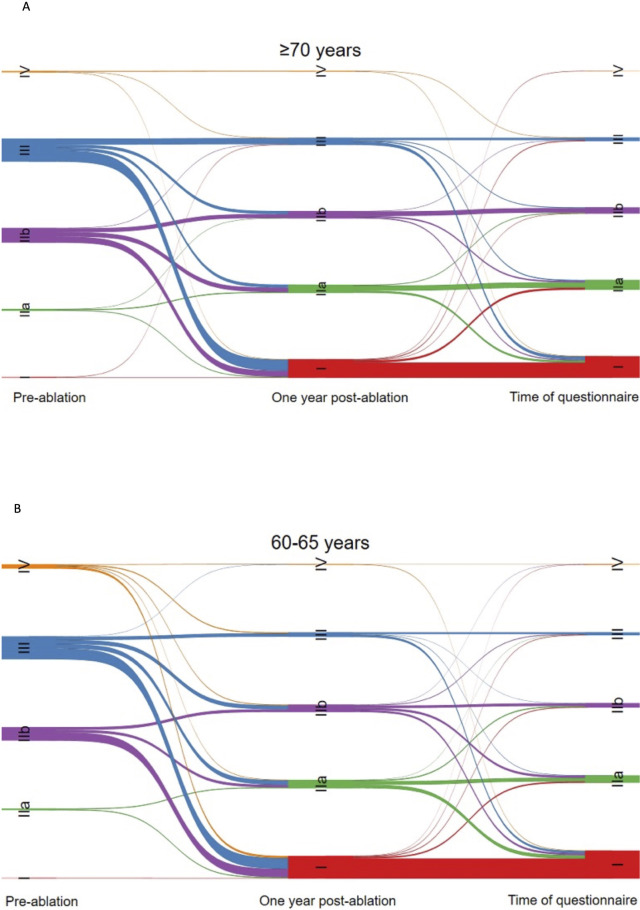
**(A)** mEHRA symptom development in the older cohort. Representation of progression of symptoms for each patient (every thin line) from one timepoint to the next. The broader the line, the more patients with the same development; **(B)** mEHRA symptom development in the younger cohort.

Symptom development was assessed as change in mEHRA. There was a significant reduction in the score from pre-ablation to one-year post-ablation within both age subgroups (≥70: ΔmEHRA −1.42 ± 1.29, *p* < 0.001; 60–65: ΔmEHRA −1.78 ± 1.17, *p* < 0.001) (Figures 2B, 3A,B). The improvement was significantly greater among patients aged 60–65 (*p* = 0.021).

A further reduction in symptom severity was reported between one year post-ablation and the time of the questionnaire (≥70: *p* = 0.010, 60–65: *p* = 0.008), with no significant difference in the change between the groups [0 (0–0) vs. 0 (0–0), *p* = 0.87]. The change from pre-ablation to the time of the questionnaire was significant in both subgroups (both *p* < 0.001) and significantly smaller among patients ≥70 years compared to those aged 60–65 years [2 (1–3) vs. 2 (1–3), *p* = 0.016].

Exploratory Spearman correlation analyses showed no significant association between time from ablation to questionnaire completion and change in mEHRA score from baseline to follow-up, either in the overall cohort (rho = −0.054, *p* = 0.33), or when stratified by age group (≥70 years: rho = −0.007, *p* = 0.94, 60–65 years: rho = −0.090, *p* = 0.20).

### 3.3 Arrhythmia burden

**Figure 4:**
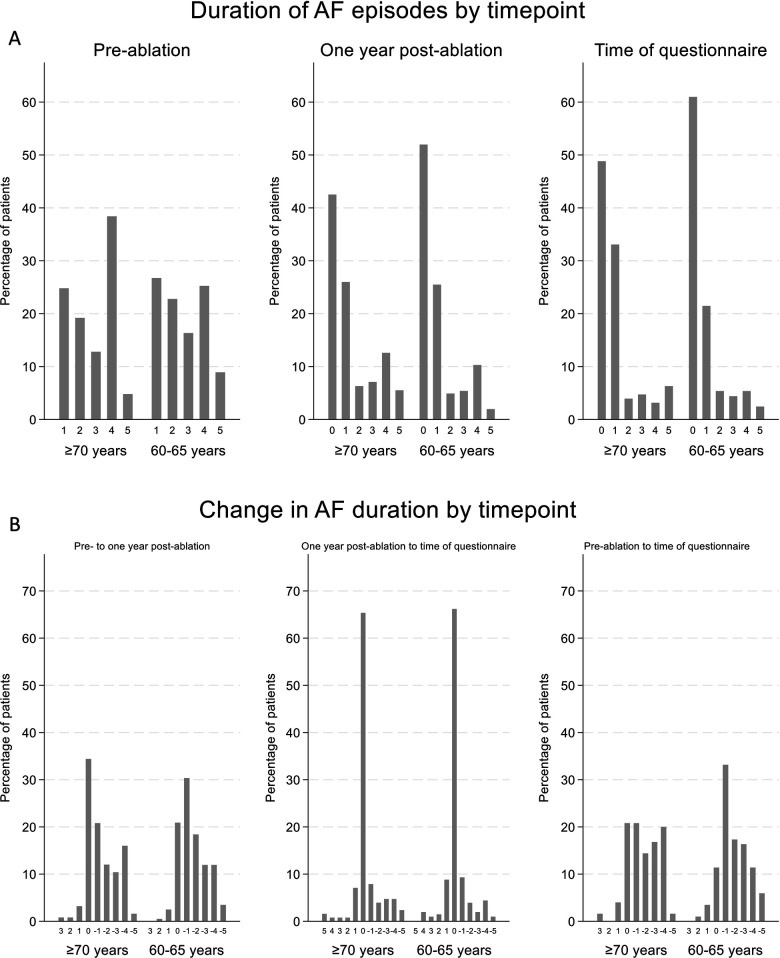
**(A)** Duration of AF episodes by timepoint. Arrhythmia burden was assessed using a five-point scale based on the duration and termination of AF episodes: 0 = no AF episodes; 1 = self-terminating episodes lasting <24 h; 2 = self-terminating episodes lasting >24 h; 3 = termination requiring pharmacological cardioversion; 4 = termination requiring electrical cardioversion; and 5 = chronic AF, indicating a shared decision between the patient and physician to forgo further attempts at rhythm control; **(B)** Change in duration of AF episodes by timepoint. Change in burden assessed using the same five-point scale. Example: a value of −2 can for instance represent a change from a score of 4 to a score of 2.

The grading of arrhythmia burden was not significantly different at baseline or at the time of the questionnaire (*p* = 0.069), but the older cohort reported significantly shorter duration of AF episodes compared to the younger cohort one-year post-ablation (*p* = 0.046) (Figure 4A). Both cohorts showed significant reductions in AF episode duration one-year post-ablation and at follow-up compared to baseline (*p* < 0.001). The duration also decreased from one-year post-ablation to the time of the questionnaire (≥70 years: *p* = 0.010; 60–65 years: *p* = 0.048) (Figure 4B).

### 3.4 Procedures, acute outcomes and complications

The two study groups differed in ablation type, with the older cohort more often receiving RF ablation (Table 2). Ablation type was not significantly associated with recurrence in either group (*p* = 0.79 and *p* = 0.45, respectively). The overall procedure time was longer in the older cohort, likely reflecting the higher portion of RF ablations in this group. When stratified by ablation modality, no significant difference in procedure duration was observed for cryoballoon procedures, whereas RF procedures were longer in the younger cohort. Other procedural characteristics were broadly similar between the groups. One of the patients in the younger cohort was found deceased at home the day after discharge post CA. Due to the temporal association with the procedure, the event was reported descriptively; however, it was not classified as a procedural complication, as the cause of death remained unknown despite autopsy.

**Table 2:** Procedural outcomes.

	≥70 years (*N* = 132)		60–65 years (*N* = 212)		
Procedural parameter	n	Value	n	Value	*p*-value
Ablation type	132		212		
Cryo (%)		39 (30)		116 (55)	<0.001*
RF (%)		93 (70)		96 (45)	<0.001*
Procedure time, min (IQR)	130	183.5 (139.0–228.0)	209	144.0 (103.0–218.0)	0.003*
Radiation time, min (IQR)	129	15.0 (9.9–24.0)	202	16.9 (11.6–28.0)	0.11
Radiation dose, uGym2 (IQR)	130	694.1 (290.0–1500.0)	200	1110.5 (571.5–2037.0)	<0.001*
RF/cryo time, sec (IQR)	117	2136.0 (1687.0–2615.0)	172	1960.5 (1547.5–2545.5)	0.20
RF applications (IQR)	28	96.0 (80.0–107.5)	64	109.0 (92.0–128.0)	0.039*
CTIB (%)	132	11 (8)	212	17 (8)	0.92
General anaesthesia (%)	132	1 (1)	212	0 (0)	0.38
Acute success (%)	132	131 (99)	212	208 (98)	0.65
Length of stay, days (IQR)	132	2.0 (2.0–2.0)	212	2.0 (2.0–2.0)	0.56
Complications (%)	132	7 (5)	212	14 (7)	0.62
Type of complication, n					0.95
Hematoma		2		6	0.72
Pericardial effusion		1		1	1.00
Pericarditis		0		1	1.00
Tamponade		2		4	1.00
TIA		1		0	0.38
Stroke		1		1	1.00
PM-requiring bradycardia		0		1	1.00
Death of unknown cause >24 h after procedure^†^		0		1	1.00

RF, radiofrequency; CTIB, cavotricuspid isthmus block; PM, pacemaker; TIA, transient ischemic attack.

* *p* < 0.05.

^†^ One patient was found dead at home a day after discharge, with unknown cause of death.

The adjacent n columns indicate the number of patients with available data for each variable. Continuous variables are presented as median (IQR) or mean (SD), and categorical variables as n (%) or n, as indicated.

### 3.5 Follow-up parameters

In the older cohort, more patients reported AF recurrence after CA, compared to the younger cohort (Table 3). This association was attenuated when comparing only recurrence reported as documented in medical records. The reported recurrence rate was also significantly higher among the patients aged ≥70 years at one-year post-ablation. There was no significant difference in time to recurrence in the two groups, and no difference in the number of patients who underwent a second ablation. Three patients in the younger cohort underwent a third ablation.

**Table 3:** Follow-up parameters.

	≥70 years (*N* = 132)		60–65 years (*N* = 212)		
Follow-up parameter	n	Value	n	Value	*p*-value
Recurrence (%)	130	76 (58.5)	208	98 (47.1)	0.042*
Documented recurrence (%)	130	70 (53.8)	208	90 (43.3)	0.058
1-year recurrence (%)	130	61 (46.9)	208	71 (34.1)	0.019*
Time to recurrence, months (IQR)	71	6 (3–12)	89	6 (4–17)	0.11
Documentation of recurrence	76		98		
ECG/Holter/loop recorder(%)		68 (89.5)		84 (85.7)	
PM (%)		1 (1.3)		0	
Smartwatch (%)		0		6 (6.1)	
BP monitor (%)		1 (1.3)		0	
Undocumented (%)		6 (7.9)		8 (8.1)	
Redo ablation (%)		24 (18.2)		39 (18.4)	0.96
Use of antiarrhythmic drugs					
1 y (%)	123	96 (72.7)	204	94 (44.3)	0.001*
Now (%)	124	96 (72.7)	199	115 (54.2)	0.001*
Use of anticoagulants					
1 y (%)	128	125 (94.7)	205	172 (81.1)	<0.001*
Now (%)	127	126 (95.5)	203	169 (79.7)	<0.001*

ECG, electrocardiogram; PM, pacemaker; BP, blood pressure.

* *p* < 0.05.

The adjacent n columns indicate the number of patients with available data for each variable. Continuous variables are presented as median (IQR) or mean (SD), and categorical variables as n (%) or n, as indicated.

At the time of follow-up, both patient groups showed significant reductions in AAD use compared to baseline (both *p* < 0.001). A higher percentage of the older cohort were still using AADs at both follow-up timepoints (Table 3). For the younger cohort, we observed a nominal increase from one-year post-ablation to the time of the questionnaire in the proportion of patients taking AADs, but this increase did not reach statistical significance. The use of anticoagulant medical therapy was also significantly reduced after one year in both groups (both *p* < 0.001). Neither group experienced further significant reduction in drug use until the time of the questionnaire.

## 4 Discussion

We evaluated clinical outcomes and PROMs in patients aged ≥70 years undergoing first-time CA for AF and compared them with patients aged 60–65 years. This real-world cohort included all consenting or deceased patients with no exclusion criteria. The two age groups were comparable at baseline, except for differences in BMI, prevalence of hypertension, and systolic blood pressure at admission. Symptom reduction, assessed using the mEHRA scale, was significant in both groups. Although the younger patients reported a larger absolute change in unadjusted analyses, a higher proportion of the ≥70 group achieved a reduction in mEHRA scores at follow-up. Furthermore, the older cohort reported shorter duration of AF episodes one-year post-ablation. However, unadjusted analyses suggested higher arrhythmia recurrence rates among older patients, and they more often remained on antiarrhythmic drugs and anticoagulant therapy.

### 4.1 PROMs vs. recurrence

Our findings illustrate a clinically important distinction between rhythm-defined recurrence and patient-perceived treatment benefit. The reported lower symptom severity scores in the older group suggest that many considered the treatment worthwhile even if AF formally recurred. These results reinforce the value of incorporating PROMs when assessing therapeutic efficacy (6, 9–11).

### 4.2 Age and benefit from CA for AF

Compared to medical therapy, CA is more effective at maintaining sinus rhythm and improving quality of life in older patients but carries a higher risk of procedure-related complications, including stroke or transient ischemic attack (12). When considering CA for a broader population of older patients, it is essential to evaluate not only recurrence rates, but also symptom burden and patient-reported outcomes, and to balance these benefits against procedural risks.

A key finding of our study is that both age groups reported significant improvement in symptom scores. Although younger patients showed a greater absolute reduction, a greater proportion of those ≥70 years recorded lower final mEHRA scores, suggesting comparable—or even greater—perceived benefit. This observation is consistent with a previous study reporting quality of life outcomes following CA for AF, where older patients exhibited scores comparable to those of younger patients (13).

We found no significant age-related difference in complication rates, contrasting previous meta-analyses that reported higher complication rates—mostly minor—in older adults (2–5). Notably, three of these studies applied a ≥75-year cutoff, thereby focusing on an older population than the older cohort in our study. This should nevertheless be interpreted cautiously, as patients accepted for CA likely represent a selected group of relatively healthy older individuals. We did not distinguish between minor and major complications because the low event numbers precluded reliable subgroup analysis. Furthermore, the low event numbers limited statistical power for safety comparisons.

Reported AF recurrence was more frequent in the older group in unadjusted analyses. Potential confounders include, but are not limited to, surveillance, medication use, AF substrate, and ablation type. Without formal adjustment, this difference should be interpreted as exploratory. Recurrence rates in both groups were higher than in several previous reports, including those reporting similar recurrence rates across age groups (2–5). The inclusion of patient-reported recurrence in our definition may partly explain this discrepancy.

### 4.3 Clinical implications

Our findings highlight the importance of patient-reported symptoms alongside conventional clinical endpoints in selected older patients undergoing CA for AF. In this population, treatment benefit may not be fully reflected by recurrence status alone. As maintenance of functional status is often an important treatment priority in older patients (7–9), symptom burden should be carefully considered when weighing the potential benefits of treatment against procedural risks. Substantial and clinically meaningful symptom improvement, low overall symptom burden at long-term follow-up—even in the presence of recurrence—and low complication rates support a patient-centered approach to treatment evaluation.

### 4.4 Limitations

This was a retrospective single-center study, and the older cohort represents a selected population of relatively healthy patients referred and accepted for ablation. The findings should therefore not be generalized to all patients aged ≥70 years with AF. At the same time, we included all eligible patients undergoing first-time ablation within the selected age groups during the study period, which supports the real-world character of the cohort.

The PROM data are subject to limitations inherent to retrospective questionnaire-based assessment, including recall bias and potential placebo effects. In particular, the pre-ablation and one-year post-ablation assessments were obtained retrospectively and are therefore subject to notable uncertainty regarding the accuracy of recalled symptom burden. Furthermore, selection bias may have been introduced if individuals who were dissatisfied with the procedure were more likely to participate. In addition, questionnaire items other than mEHRA score were not formally validated, although they were based on clinically relevant items used in structured follow-up at our center. Missing questionnaire responses were clarified by telephone when possible, and remaining missing data were handled using available-case analysis.

Older patients more often underwent RF ablation than cryoballoon ablation. This partly reflected operator preference for a strict CLOSE-guided RF approach and for more detailed electroanatomical assessment of atrial substrate, including low-voltage areas, in selected older individuals. As the study period predates the widespread adoption of pulsed field ablation, RF and cryoballoon ablation represented the available modalities. However, the non-random distribution of ablation modality remains a potential source of confounding when comparing procedural and follow-up outcomes between groups.

Finally, the older cohort remained more frequently on antiarrhythmic drugs during follow-up, which may have influenced both symptom burden and recurrence patterns. The relatively small sample size and low number of complications also limited statistical power for detecting differences in adverse events.

## 5 Conclusion

In this real-world cohort of patients undergoing first-time CA for AF, selected patients aged ≥70 years reported clinically meaningful symptom improvement and no statistically significant difference in complication rates compared with patients aged 60–65 years. Despite higher reported recurrence rates, there was a greater proportion of older patients reporting symptom improvement, and the older patients reported shorter AF episodes one-year post-ablation. These findings highlight the importance of PROMs alongside rhythm outcomes when evaluating treatment benefit in older patients. Larger prospective multicenter trials incorporating frailty assessment, PROMs, and continuous rhythm monitoring are warranted to assess safety and comparative effectiveness.

## Data Availability

Due to ethical and privacy restrictions related to sensitive patient data, the datasets generated and/or analyzed during the current study are not publicly available. Data may be made available from the corresponding author upon reasonable request and subject to relevant ethical and institutional approvals.
