# Supplementary material for: Patient-reported and clinical outcomes after first-time atrial fibrillation ablation in older patients: a real-world retrospective single-center study
Source: Front Cardiovasc Med. 2026 Jul 8;13:1841603. doi: 10.3389/fcvm.2026.1841603 (PMC13388553; doi:10.3389/fcvm.2026.1841603)
Supplement: Supplementary file 1 [file Datasheet1.docx]

# Supplementary material

## 1. Supplementary Material S1, Questionnaires

Pasientnavn: «Fullt navn» . Fødselsnummer: «Fødselsnummer»

**Oppfølging av pasienter etter ablasjon av atrieflimmer (hjerteflimmer)**

Vi ønsker å vite mer om hvordan det har gått med deg etter ablasjonsbehandlingen på Rikshospitalet «dato». ***Dine erfaringer er viktige for oss, og vi håper du vil ta deg tid til å fylle ut dette spørreskjemaet!*** Det endelige målet er å tilby bedre behandling til pasienter med atrieflimmer.

Dersom det er spørsmål det er vanskelig å forstå kan du krysse av for at vi kontakter deg pr. telefon (nederst i spørreskjemaet eller på samtykkeskjemaet).

**Sett en ring rundt det alternativet du synes passer best**

1. **Hvis en ser bort fra de første 3 månedene, er det hos lege eller andre registrert atrieflimmer hos deg etter ablasjonen? (registrert med EKG eller annen måte)**

1. Ja 2. Nei

Hvis ja, hvor lang tid etter ablasjonen var det første flimmeranfallet (utenom de første 3mnd)?

Svar: _____________________________________________________________

1. **Hvordan er tilbakefall dokumentert? Hos lege/sykehus, på EKG, døgnregistrering av EKG, smartklokke, annet?**

Svar: _____________________________________________________________

1. **Hvilken lege/hvilket sykehus har du vært til kontroll hos etter ablasjonen?**

Svar: ____________________________________________________________

1. **Har du gjennomgått ny ablasjon av hjerteflimmeren etter ablasjonsbehandlingen den «dato» ?**

1. Ja 2. Nei

Hvis ja, hvor og når:________________________________________________________

1. **Angi i hvilken grad symptomer som skyldtes hjerteflimmer påvirket(er) din daglige aktivitet før ablasjonen, 1 år etter og nå. Bruk følgende gradering (1-5):
   1. Ingen symptomer
   2. Milde symptomer**: Ingen påvirkning av normal daglig aktivitet og symptomene er i liten grad plagsomme
   **3. Moderate symptomer**: Ingen påvirkning av normal daglig aktivitet, men symptomene er plagsomme
   **4. Alvorlige symptomer**: Normal daglig aktivitet er påvirket
   **5. Invalidiserende symptomer**: Normal daglig aktivitet er ikke lenger mulig

Sett ring rundt tallet som best beskriver situasjonen:

| **Grad av symptom** | **Før ablasjonen** | **1 år etter ablasjonen** | **Nå** |
| --- | --- | --- | --- |
| **Ingen** | 1 | 1 | 1 |
| **Mild** | 2 | 2 | 2 |
| **Moderat** | 3 | 3 | 3 |
| **Alvorlig** | 4 | 4 | 4 |
| **Invalidiserende** | 5 | 5 | 5 |

1. **Hvordan vil du beskrive dine typiske anfall med atrieflimmer?**

**1. Korte selvbegrensende:** Varighet under 24 timer – stopper uten ekstra behandling. **2. Lange selvbegrensende**: Varighet over 24 timer – stopper uten ekstra behandling
**3. Vedvarende til medikamentell konvertering**: Vedvarende – til behandling med ekstra medikamenter
**4. Vedvarende til elektrokonvertering**: Vedvarende – til elektrisk konvertering i narkose
**5. Kronisk**: Vedvarende hjerteflimmer – man har gitt opp forsøk på normalisering av hjerterytmen

Sett ring rundt tallet som best beskriver anfallene:

| **Anfallstype** | **Før ablasjonen** | **1 år etter ablasjonen** | **Nå** |
| --- | --- | --- | --- |
| **Ingen anfall** |  | 0 | 0 |
| **Korte selvbegrensende** | 1 | 1 | 1 |
| **Lange selvbegrensende** | 2 | 2 | 2 |
| **Vedvarende til medikamentell konvertering** | 3 | 3 | 3 |
| **Vedvarende til elektrokonvertering** | 4 | 4 | 4 |
| **Kronisk** | 5 | 5 | 5 |

1. **Hvilke medisiner har du brukt mot atrieflimmer før ablasjonen, 1 år etter og nå?**

Sett ring rundt alle medikamentene du brukte/bruker

| **Før ablasjonen** | **1 år etter ablasjonen** | **Nå** |
| --- | --- | --- |
| MEDISINER MOT ATRIEFLIMMER | | |
| Ingen | Ingen | Ingen |
| Metoprolol/Bisoprolol | Metoprolol/Bisoprolol | Metoprolol/Bisoprolol |
| Sotacor/Sotalol | Sotacor/Sotalol | Sotacor/Sotalol |
| Multaq | Multaq | Multaq |
| Cordarone | Cordarone | Cordarone |
| Tambocor | Tambocor | Tambocor |
| Andre mot rytmeforstyrrelse | Andre mot rytmeforstyrrelse | Andre mot rytmeforstyrrelse |
| BLODFORTYNNENDE MEDISINER | | |
| Ingen | Ingen | Ingen |
| Marevan | Marevan | Marevan |
| Eliquis | Eliquis | Eliquis |
| Xarelto | Xarelto | Xarelto |
| Lixiana | Lixiana | Lixiana |
| Pradaxa | Pradaxa | Pradaxa |
| Andre blodfortynnende | Andre blodfortynnende | Andre blodfortynnende |

Om du har satt ring rundt «Andre mot rytmeforstyrrelse» - Hvilke, og når?

Svar: ______________________________________________________________________

Om du har satt ring rundt «Andre blodfortynnende» - Hvilke, og når?

Svar: ______________________________________________________________________

1. **Fikk du noen komplikasjoner/problemer som følge av ablasjonsbehandlingen?**

1. Ja 2. Nei

Hvis du svarte ja på spm 8, hva slags komplikasjoner/problemer og når?

___________________________________________________________________________

___________________________________________________________________________

Hvis du har søkt/fått helsehjelp for komplikasjonene et annet sted enn Rikshospitalet, hvor? Samtykker du til at vi henter helseopplysninger om deg fra dette stedet?

Sted: _____________________________ 1. Samtykker 2. Samtykker ikke

1. **Har du andre kommentarer vedrørende sykdommen, medisinene du har brukt, behandlingen på Rikshospitalet eller oppfølgingen etter behandlingen?**

Skriv her:

___________________________________________________________________________

___________________________________________________________________________

___________________________________________________________________________

___________________________________________________________________________

___________________________________________________________________________

**Ønsker du å at vi kontakter deg over telefon for å svare på spørreskjemaet?**

Ja Hvilket telefonnummer kan vi nå deg på? Tlf: _____________________________

Dato: __________________________________________

Signatur:________________________________________

Signert spørreskjema (dette brevet) og samtykkeerklæringen returneres i vedlagte ferdig adresserte og frankerte konvolutt til:

**Overlege Erik Lyseggen**

**Kardiologisk avdeling, Rikshospitalet, Oslo Universitetssykehus
Postboks 4950 Nydalen
0424 Oslo**

TUSEN TAKK FOR HJELPEN!

## Supplementary Table S2

|  | ≥70y (N = 132) | | 60-65y (N = 212) | | p-value |
| --- | --- | --- | --- | --- | --- |
|  | N |  | N |  |  |
| **Age at procedure, years** | 132 | 73.0 (71.0-75.0) | 212 | 63.0 (61.0-64.0) | <0.001* |
| **Gender, Male (%)** | 132 | 85 (64) | 212 | 154 (73) | 0.11 |
| **Height, m (IQR)** | 131 | 1.8 (1.7-1.8) | 211 | 1.8 (1.7-1.9) | 0.017* |
| **Weight, kg** | 131 | 81.7 (12.2) | 211 | 89.0 (14.3) | <0.001* |
| **BMI** | 131 | 26.0 (3.4) | 211 | 27.6 (3.8) | <0.001* |
| **Type of AF** | 131 |  | 209 |  | 0.15 |
| **Paroxysmal (%)** |  | 95 (72) |  | 168 (79) | 0.12 |
| **Paroxysmal persistent (%)** |  | 6 (5 ) |  | 11 (5 ) | 0.79 |
| **Persistent (%)** |  | 28 (21) |  | 24 (11) | 0.13 |
| **Long-standing persistent (%)** |  | 2 (2) |  | 6 (3) | 0.72 |
| **AF duration, years (IQR)** | 118 | 4.0 (2.0-9.0) | 191 | 3.0 (1.2-7.0) | 0.035 |
| **CHA2DS2VA (IQR)** | 132 | 2.0 (1.0-3.0) | 212 | 1.0 (0.0-1.0) | <0.001* |
| **Heart failure (%)** | 132 | 8 (6) | 212 | 20 (9) | 0.27 |
| **Hypertension (%)** | 132 | 62 (47) | 212 | 73 (34) | 0.021* |
| **Diabetes Mellitus (%)** | 132 | 7 (5) | 212 | 13 (6) | 0.75 |
| **History of TIA/stroke/TE (%)** | 132 | 7 (5) | 212 | 21 (10) | 0.13 |
| **Stroke (%)** |  | 2 (2) |  | 5 (2) | 0.59 |
| **TIA (%)** |  | 3 (2) |  | 8 (4) | 0.44 |
| **TE (%)** |  | 2 (2) |  | 8 (4) | 0.23 |
| **Vascular disease (%)** | 132 | 25 (19) | 212 | 26 (12) | 0.090 |
| **CAD (%)** |  | 24 (18) |  | 25 (12) | 0.099 |
| **MI (%)** |  | 13 (10) |  | 14 (7) | 0.28 |
| **PAD (%)** |  | 1 (1) |  | 0 (0) | 0.38 |
| **History of PCI/CABG (%)** | 132 | 20 (15) | 212 | 21 (10) | 0.14 |
| **Systolic BP at admission, mm Hg (IQR)** | 125 | 140.0 (131.0-159.0) | 197 | 135.0 (121.0-150.0) | 0.002* |
| **Diastolic BP at admission, mm Hg (IQR)** | 125 | 79.0 (71.0-85.0) | 197 | 78.0 (72.0-84.0) | 0.96 |
| **Cardiomyopathy (%)** | 132 | 3 (2) | 212 | 10 (5) | 0.38 |
| **TIHF (%)** | 132 | 4 (3) | 212 | 9 (4) | 0.77 |
| **Sleep apnea (%)** | 132 | 12 (9) | 212 | 28 (13) | 0.25 |
| **Atrial flutter (%)** | 132 | 19 (14) | 212 | 45 (21) | 0.11 |
| **PM (%)** | 132 | 6 (5) | 212 | 4 (2) | 0.19 |
| **CRT-P (%)** | 132 | 0 (0) | 212 | 2 (1) | 0.53 |
| **ICD (%)** | 132 | 1 (1) | 212 | 6 (3) | 0.26 |
| **Dyslipidemia (%)** | 132 | 16 (12) | 212 | 23 (11) | 0.72 |
| **COPD (%)** | 132 | 3 (2) | 212 | 4 (2) | 1.00 |
| **Pulmonary hypertension (%)** | 132 | 2 (2) | 212 | 2 (1) | 0.64 |
| **CKD (%)** | 132 | 8 (6) | 212 | 5 (2) | 0.080 |
| **Other comorbidities (%)** | 132 | 91 (69) | 212 | 128 (60) | 0.11 |
| **EF % (IQR)** | 75 | 55.0 (50.0-60.0) | 115 | 55.0 (51.0-60.0) | 0.74 |
| **LVEDD, cm (SD)** | 132 | 5.1 (0.5) | 204 | 5.2 (0.7) | 0.28 |
| **LAD, cm (IQR)** | 56 | 4.2 (3.8-4.7) | 94 | 4.4 (3.9-4.8) | 0.63 |
| **LAA, cm2 (IQR)** | 128 | 24.2 (21.0-28.0) | 196 | 24.4 (21.0-28.0) | 0.70 |
| **LAVI, mL/m2 (IQR)** | 90 | 40.5 (32.0-48.0) | 131 | 37.0 (31.0-47.0) | 0.19 |
| **RAA, cm2 (IQR)** | 71 | 20.0 (16.0-23.0) | 100 | 20.0 (17.0-24.0) | 0.39 |
| **Degree of MR (IQR)** | 128 | 0.5 (0.5-1.0) | 188 | 0.5 (0.5-1.0) | 0.081 |
| **MR (%)** | 130 | 66 (51) | 206 | 83 (40) | 0.071 |
| **Other valve disease (%)** | 132 | 13 (10) | 207 | 7 (3) | 0.014* |
| **Use of antiarrhytmics (%)** | 132 | 186 (88) | 212 | 119 (90) | 0.49 |
| **Metoprolol/Bisoprolol (%)** |  | 75 (57) |  | 126 (59) | 0.63 |
| **Sotalol (%)** |  | 4 (3) |  | 3 (1) | 0.30 |
| **Dronedarone (%)** |  | 26 (20) |  | 36 (17) | 0.52 |
| **Amiodarone (%)** |  | 33 (25) |  | 44 (21) | 0.36 |
| **Flecainide (%)** |  | 28 (21) |  | 45 (21) | 1.00 |
| **Other (%)** |  | 0 (0) |  | 1 (≈ 0) | 0.73 |
| **Use of anticoagulants (%)** | 132 | 131 (99.2) | 212 | 207 (97.6) | 0.270 |
| **Marevan (%)** |  | 5 (4) |  | 9 (4) | 0.84 |
| **Apixaban (%)** |  | 92 (70) |  | 132 (62) | 0.13 |
| **Rivaroxaban (%)** |  | 28 (21) |  | 47 (22) | 0.83 |
| **Edoxaban (%)** |  | 2 (2) |  | 9 (4) | 0.16 |
| **Dabigatran etexilate (%)** |  | 5 (4) |  | 9 (4) | 0.84 |
| **Other (%)** |  | 7 (5) |  | 6 (3) | 0.24 |

**Supplementary Table S2: detailed baseline characteristics.** The adjacent n columns indicate the number of patients with available data for each variable. Continuous variables are presented as median (IQR) or mean (SD), and categorical variables as n (%) or n, as indicated. *p <0.05. Abbreviations: AF, atrial fibrillation; BMI, body mass index; BP, blood pressure; CABG, coronary artery bypass graft surgery; CAD, coronary artery disease; CHA2DS2VA, Congestive heart failure, Hypertension, Age ≥75 years (doubled), Diabetes mellitus, prior Stroke or TIA or thromboembolism (doubled), Vascular disease, Age 65 to 74 years; CKD, chronic kidney disease; COPD, chronic obstructive pulmonary disease; CRT-P, cardiac resynchronization therapy pacemaker; EF, ejection fraction; ICD, implantable cardioverter-defibrillator; LAA, left atrial area; LAD, left atrial diameter; LAVI, left atrial volume index; LVEDD, left ventricular end-diastolic diameter; MI, myocardial infarction; MR, mitral valve regurgitation; PAD, peripheral artery disease; PCI, percutaneous coronary intervention; PM, pacemaker; RAA, right atrial area; TE, thromboembolism; TIA, transient ischemic attack; TIHF, tachycardia-induced heart failure.
